# Driving mechanism of subjective cognition on farmers’ adoption behavior of straw returning technology: Evidence from rice and wheat producing provinces in China

**DOI:** 10.3389/fpsyg.2022.922889

**Published:** 2022-08-02

**Authors:** Zhong Ren, Kaiyang Zhong

**Affiliations:** ^1^Business School, Shandong Normal University, Jinan, China; ^2^School of Economic Information Engineering, Southwestern University of Finance and Economics, Chengdu, China

**Keywords:** straw returning, subjective cognition, TPB, SEM, straw returning technology

## Abstract

Straw burning is one of the important causes of environmental pollution in rural China. As an important green production technology, straw returning is beneficial to the improvement of rural environment and the sustainable development of agriculture. Based on the improved planned behavior theory, taking the survey data of 788 farmers in Shandong, Henan, Hubei, and Hunan provinces as samples, this paper uses a multi-group structural equation model to explore the driving mechanism of subjective cognition on the adoption behavior of farmers’ straw returning technology. The results show that behavioral attitude, subjective norm, and perceived behavioral control, which represent subjective cognition, all have significant driving effects on farmers’ intention to adopt straw returning technology. Behavioral intention plays a mediating role in the process of subjective cognition driving farmers’ adoption behavior of straw returning technology. Government support has a moderating role in the path from farmers’ behavioral intention to behavioral response. The subjective cognition of different types of farmers has a significant driving effect on the adoption intention of straw returning technology, but the driving strength weakens with the increase of the degree of farmers’ concurrent occupation. This study provides guidance for improving the government’s straw returning policy and regulating straw returning behavior.

## Introduction

**Figure 1:**
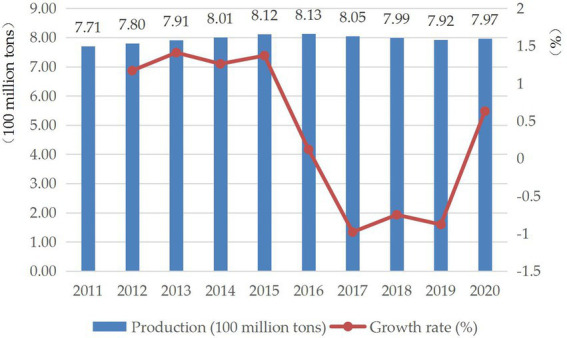
Production and growth rate of straw in China from 2011 to 2020.

Crop straw is an agricultural biomass resource with high utilization value. According to “Development Report of China’s Straw Industry in 2021,” China produces about 800 million tons of straw every year (Figure 1), accounting for one-third of the global total, and the total amount of straw resources is considerable (Development Report of China's Straw Industry in, 2021). For a long time, China’s crop straw was mainly used for living fuel and livestock raising (Lu, 2015), but since the 1980s, with the development of economic and social transformation, the function of direct utilization of straw is gradually weakened, and open burning has become a common method of straw treatment because it is convenient and fast, and meets the time requirements of double and triple crop systems in many areas of China (Yu et al., 2017; Ren et al., 2019). However, open burning of straw will release a large amount of pollutants such as nitrogen oxides (NO_X_) and sulfur oxides (SO_2_) into the atmosphere, aggravating the formation of smog and harming human health (Wen et al., 2020). The haze from burning will also affect the operation of public transportation such as highways and aviation, and even cause fires, causing serious economic losses and social impacts (Jiang et al., 2019).

As a simple and low-cost conservation tillage technology, straw returning can improve soil fertility, increase crop yield, and significantly reduce the negative externalities caused by open burning of straws (Wang et al., 2012; Hou et al., 2019). It is an effective way of resource utilization of agricultural waste. Therefore, the Chinese government attaches great importance to the promotion of straw returning technology. In 2008, the State Council issued the “Opinions on Accelerating the Comprehensive Utilization of Crop Straw,” which proposed to promote the technology of direct straw returning to the field by adopting operation subsidies and technical training, and adopting strict supervision and punishment measures for open burning of straw (State Council, 2008). In 2019, the Ministry of Agriculture and Rural Areas issued the “Notice on Comprehensively Utilization of Straw,” emphasizing that the comprehensive utilization of straw should be accelerated nationwide (Ministry of Agriculture and Rural Areas, 2019). Regrettably, the straw returning policy has been promoted in China for many years, but with little success. Farmers have a cold attitude toward straw returning, and their enthusiasm for participation is not high. The straw burning problem has not been effectively solved (Li et al., 2018; Lu et al., 2020). Therefore, how to improve the application level of straw returning to the field and reducing the environmental pollution caused by straw burning is a major practical problem faced by the sustainable development of China’s rural areas.

Farmers are the ultimate executors and direct beneficiaries of straw returning technology, and the widespread application of straw returning technology largely depends on farmers’ acceptance of the technology. Therefore, it is of great significance to explore the decision-making mechanism of farmers’ adoption of straw returning technology to improve the application level of straw returning to the field (Wang et al., 2022). Numerous studies have found that economic factors have an important impact on farmers’ adoption behavior of straw returning technology. Huang et al. (2019) estimated through the conditional value method that the cost of farmers in Jiangsu Province, China, after adopting the straw returning technology would increase by 743 RMB/ha, and all of them would be borne by individuals, which reduced farmers’ intention to adopt, and the government subsidy standard raising will help to increase farmers’ intention to adopt. Yang et al. (2020) believed that the high cost and uncertain benefits of straw returning to the field are important reasons for farmers’ reluctance to adopt. Hou et al. (2019) found that increasing subsidies for straw shredders can motivate farmers to adopt straw returning technology and reduce straw burning. However, economic factors are not the only factors that affect farmers’ adoption of straw returning technology, and the external environment also has an important impact. Seglah et al. (2020) investigated the northern region of Ghana and found that large-scale straw burning hinders the effective use of straw resources, which is closely related to the lack of agricultural extension training and the lack of government support for prohibiting field burning of straw. Jiang et al. (2020) research findings interactions between media channels and social interactions facilitate the adoption of straw return by farmers and reinforce each other. Zheng et al. (2022) found that the use of the Internet can increase the probability of farmers adopting straw returning technology by 0.155. In addition, demographic characteristics such as education level, labor force, land scale, and income level have also been confirmed to have an important impact on the adoption of straw returning technology by farmers (Quan et al., 2011; Mao et al., 2021; Zhong et al., 2021a).

Judging from the existing research content, most of the existing research focuses on analyzing the factors affecting farmers’ adoption behavior of straw returning technology from the aspects of economic factors, external environment, and demographic characteristics. These studies are based on the assumption that the objective reality faced by farmers is the basis for their behavioral decisions. However, whether or not farmers adopt the behavioral decision of straw returning technology is the best choice made under the combined influence of rational and emotional based on their subjective cognition. Their behavioral decisions are not only restricted by objective and realistic conditions, but also affected by subjective cognition formed under a specific social and cultural background (Liu and Luo, 2018), especially the aggravation of pollution caused by straw burning, which also shapes farmers’ understanding of environmental problems (Lu et al., 2020). Therefore, farmers’ subjective cognitive factors have attracted more and more attention in recent years. Guo et al. (2021) analyzed that the behavioral attitude, subjective norms, perceived behavioral control, and moral responsibility of farmers have a significant positive impact on the intention to use straw resources. Li et al. (2020) found that perceived value and perceived benefit had a significant positive impact on farmers’ intention to produce green agriculture, while perceived risk had a significant negative impact. Cao et al. (2020) pointed out that farmers’ cognition of farmland protection policies can form subjective norms, which, in turn, guide their environmental protection agricultural practices.

In summary, the existing research has laid a good foundation and reference for this paper, but there are still some shortcomings: First, farmers’ adoption behavior of straw returning technology is a decision-making process from cognition to intention to response. It is necessary to understand farmers’ intention to adopt, but also to grasp the response law of converting intention into actual actions (Yan et al., 2021). Simply studying the influence of cognition on intention or behavior is difficult to grasp its inherent laws. Second, individual behavior is not only affected by intention, but also by external environmental variables such as facilitative conditions (Jeon et al., 2011). Existing studies mostly ignore the moderating role of external environmental variables in the process of converting intention into behavior. Third, existing studies generally conduct integrated research on farmers, but with the advancement of urban–rural integration in China, the continuous improvement of the degree of concurrent employment has led to the differentiation of farmers’ groups (Yuan et al., 2018; Zhong et al., 2021b), and there may be differences in the influence of different types of farmers’ subjective cognition in the adoption behavior of straw returning technology.

The main contributions of this paper are as follows: Firstly, based on the improved Theory of Planned Behavior, systematically explore the whole process of farmers’ straw returning technology adoption behavior, so as to fully grasp the inherent law of farmers’ straw returning technology adoption behavior. Secondly, the degree of concurrent employment is selected as the adjustment variable for multi-group analysis to explore the differences in the driving mechanism of straw returning technology adoption behavior by different types of farmers, so as to improve the pertinence and matching degree of policies.

This paper is organized as follows: The next section offers a conceptual frame and a number of hypotheses. It is followed by the research methods and data sources. In Section “Data analysis and empirical results,” we made an empirical analysis and test. In Section “Conclusion and suggestions,” we get our research conclusions and put forward policy suggestions.

## Theoretical analysis and research hypothesis

### Theoretical analysis

Cognitive psychology theory holds that cognition is the process by which individuals receive, process, store, and apply the acquired information. Behavioral response is a decision made by an individual based on a comprehensive analysis of his own factors and external environment based on cognitive thinking. Therefore, the generation of individual behavior depends on their cognitive ability, and different cognitive degrees will lead to different behavioral responses (Zhang et al., 2021). In order to further explore the specific influencing mechanism between cognition and behavior, this paper intends to introduce the Theory of Planned Behavior.

TPB is a modified model proposed by Ajzen based on the Theory of Reasoned Action (TRA), which is widely used to explain and predict individual behavior motivation and intention (Ajzen, 1991). The theory points out that behavioral intention is the direct factor driving behavioral response, and subjective cognition is the influencing factor driving behavioral intention. Subjective cognition can be manifested as behavioral attitude (AB), subjective norm (SN), and perceived behavioral control (PBC). Although this theory has been strongly applied in farmers’ use of improved grassland (Elahi et al., 2021), use of antimicrobials prudently (Vasquez et al., 2019), adoption of integrated pest management (Rezaei et al., 2019), and adoption of animal-friendly practices (Borges et al., 2019), it only considers the influence of individual cognition on behavioral intention, and does not introduce other external socioeconomic variables, which limits the predictive power of behavioral intention to behavioral response (Cook et al., 2016). Since then, Ajzen’s further research has shown that individual behavioral intention is not always successfully transformed into a behavioral response, and the process of converting intention into behavior is also affected by the external socioeconomic variables (Ajzen, 2011). Jeon et al. (2011) also pointed out that despite the strong intention, when there are obvious obstacles to hinder the behavior, the behavior cannot be easily realized, which further indicates that the external socioeconomic variables are particularly important in the process of behavior formation.

In the adoption behavior of farmers’ straw returning technology, behavioral response is a rational decision made by farmers based on their cognition and evaluation of economic, environmental, risk, and other factors; understanding farmers’ behavioral attitude, subjective norm, perceived behavioral control, and other cognitive constructs toward straw returning technology is a prerequisite for understanding farmers’ behavioral response; and behavioral intention plays an intermediary role between subjective cognition and behavioral response. At the same time, China’s straw returning technology mainly implements the government-led promotion mode. On the one hand, as a policymaker, the government’s support for straw returning technology affects farmers’ decision-making; On the other hand, without the government’s support, which is an important external promotion condition, farmers are constrained by the cost burden and technical difficulties of straw returning, and even if they have the intention, it is difficult to translate into actual behavior response. Based on this, this paper establishes a theoretical model of improvement planning behavior, and adds the auxiliary variable of “government support” between the behavioral intention and behavioral response of the TPB to consider the moderating effect of the external socioeconomic variable of “government support.”

### Research hypothesis

Behavioral attitude refers to an individual’s judgment of the level of liking or disliking to perform a particular behavior. Farmers’ attitudes toward the adoption of straw returning technology can be reflected by expected benefits (Meijer et al., 2015). Specifically, farmers’ cognition of the expected benefits of straw returning technology can be divided into three dimensions: economy, society, and ecology. If farmers realize that straw returning technology can increase grain output, raise income level, and obtain higher economic benefits, their behavioral attitude will be more positive. If farmers realize that straw returning technology can benefit rural development, promote social progress, and produce better social benefits, their behavioral attitude will be more positive. If farmers realize that straw returning technology can improve the ecological environment, make rational use of resources, and bring positive ecological benefits, their behavioral attitude will be more positive. To sum up, this paper measures farmers’ cognition of behavioral attitude in the adoption of straw returning technology from three dimensions of economic benefit, social benefit, and ecological benefit, and puts forward the following hypotheses:

*H1*: The behavioral attitude of farmers has a direct driving effect on the adoption intention of straw returning technology.

Subjective norm refers to the external pressure that an individual perceives when deciding whether to implement a specific behavior, which reflects the influence of important individuals or groups on individual behavioral decision-making, including two dimensions: mandatory norm and exemplary norm (Cialdini et al., 1991). Mandatory norms can be understood as farmers’ cognition of village cadres’ advocating the adoption of straw returning technology (Yu et al., 2018). The positive encouragement and strong restraint of village cadres can prompt farmers to think “I should adopt” and “I must adopt,” and then transform it into their inner intention to adopt. Exemplary norms can be understood as farmers’ cognition of the adoption of straw returning technology by relatives and friends. The recognition and positive evaluation of relatives and friends will encourage farmers to have a herd mentality, and then produce a positive adoption intention. Therefore, this paper measures farmers’ subjective norm cognition in the adoption of straw returning technology from two dimensions of mandatory norm and exemplary norm, and puts forward the following hypotheses:

*H2*: The subjective norm of farmers has a direct driving effect on the adoption intention of straw returning technology.

Perceived behavioral control refers to the individual’s perception of the difficulty of implementing a specific behavior, including two dimensions: self-efficacy and perceived difficulty (Kraft et al., 2005). Self-efficacy can be understood as farmers’ self-confidence in the technology and cost needed to adopt straw returning behavior. Perceived difficulty can be understood as farmers’ judgment on the difficulty of straw returning technology. Theoretically, the stronger the farmers’ sense of self-efficacy, the less difficult it is to perceive, and the higher their enthusiasm for adopting intentions. Therefore, this paper measures farmers’ perceived behavioral control in the adoption of straw returning technology from two dimensions of self-efficacy and perceived difficulty, and puts forward the following hypotheses:

*H3*: The perceived behavioral control of farmers has a direct driving effect on the adoption intention of straw returning technology.

Behavioral intention refers to the strength of an individual’s tendency to carry out a specific behavior. In farmers’ adoption behavior of straw returning technology, behavioral intention refers to the subjective probability of farmers’ behavioral response. Theoretically, the stronger farmers’ intention to adopt straw returning technology, the more active their practical actions will be. Behavioral attitude, subjective norm, and perceived behavioral control all indirectly drive behavioral response through behavioral intention. In addition, behavioral attitude, subjective norm, and perceived behavioral control may be correlated in pairs (Zhang et al., 2021). Based on this, puts forward the following hypotheses:

*H4*: The behavioral intention of farmers has a mediating role in the driving process of subjective cognition on the adoption of straw returning technology.

*H5*: There is an interaction effect among farmers' behavioral attitude, subjective norm, and perceived behavioral control.

Government support is divided into two aspects: policy support and technical support. If farmers feel that the government supports straw returning technology through policy and shares the successful experience of other farmers’ straw returning technology, and at the same time provides equipment, technical support, and related consultation and training, farmers’ enthusiasm for adoption will be higher, and it will be easier for them to respond to the intention transformation behavior. Therefore, government support can help farmers who have the intention but not the ability to take it into action. Based on this, puts forward the following hypotheses:

*H6*: Government support has a moderating role in the path from farmers' behavioral intention to behavioral response.

**Figure 2:**
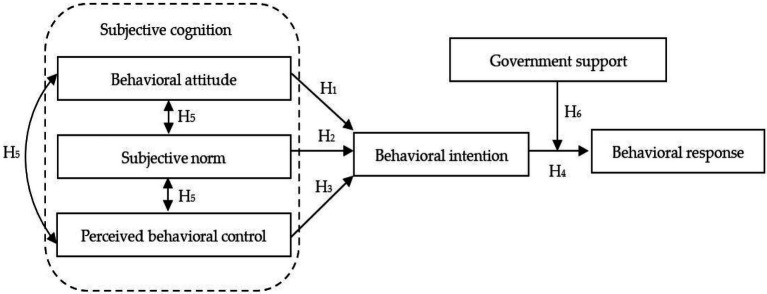
Theoretical analysis framework.

In summary, this study proposes a research model as shown in Figure 2.

## Data, variables, and model

### Data sources

**Figure 3:**
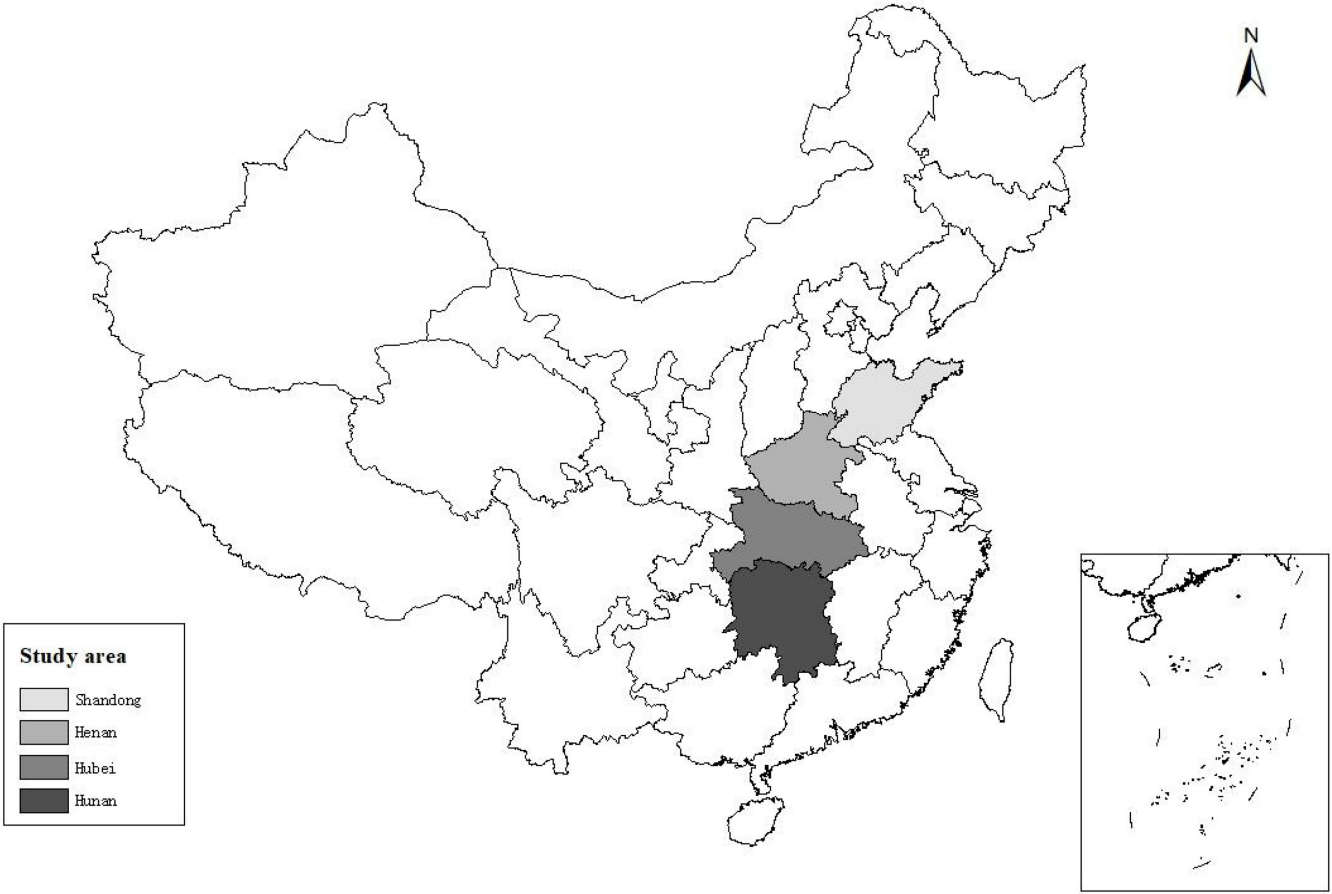
Study area.

The data used in this study came from questionnaires distributed by our research group in rural areas of Henan, Shandong, Hubei, and Hunan from June to August 2021. Henan and Shandong, located in the northern dry farming areas, are the main grain-producing areas of China’s wheat, corn, and other agricultural products, which are suitable for popularizing the technology of “returning two crops to fields.” Hubei and Hunan, located in paddy fields in the south of China, are the main rice-producing areas in China, which are suitable for popularizing the technology of returning rice straw to fields. Therefore, it is highly representative and scientific to select the above four provinces as the study areas (Figure 3).

The sample selection adopts a combination of random sampling and stratified sampling. First select two cities (counties) with large straw yield in each province, then randomly select 1–2 townships in each city (county), and then randomly select 2–3 sample villages from each township, and finally, 15–20 farmers are randomly selected from each village. Participants were explicitly informed that the questionnaire was kept confidential and that the data would be used only for research purposes. All subjects gave their informed consent for inclusion before they participated in the study. Ethical approval was obtained from the Experimental and Animal Ethics Review Board of Shandong Normal University. Face-to-face interviews were used to deeply understand the farmers and their families, subjective cognition, and straw returning adoption. A total of 840 questionnaires were distributed, 52 invalid questionnaires were excluded, and 788 valid questionnaires were obtained, with an effective rate of 93.8%.

From the perspective of sample distribution characteristics, the sample proportions of Henan, Shandong, Hubei, and Hunan are 25.1, 27.4, 22.5, and 25.0%, respectively, and the sample distribution ratios in each province are relatively close. From the perspective of individual basic characteristics, in terms of gender, male accounted for 84.6% and female accounted for 15.4%. In terms of age, farmers aged 40–60 accounted for the highest proportion (77.2%), with an average age of about 52. In terms of education level, 81.4% of the farmers’ education level is junior high school or below, and the education level is generally low. In terms of annual household income, the annual income level of most farmers is below 90,000 RMB (91.2%). The main characteristics of the samples and their distribution are shown in Table 1.

**Table 1:** Main characteristics of the sample and its distribution.

Type	Option	Quantity	Proportion	Type	Option	Quantity	Proportion
Sex	Male	667	84.6	Education level	Primary school	215	27.3
	Female	121	15.4		Junior high school	426	54.1
Age	Under 40	76	9.6		High school	106	13.5
	40–50	263	33.4		Junior college	29	3.7
	50–60	345	43.8		University and above	12	1.4
	Over 60	104	13.2	Annual income/million RMB	Under 3	81	10.2
Region	Henan	198	25.1		3–6	283	35.9
	Shandong	216	27.4		6–9	355	45.1
	Hubei	177	22.5		9–12	51	6.5
	Hunan	197	25.0		Over 12	18	2.3

### Variable definition

1. Farmers’ intention and behavior of straw returning technology. Referring to the research of Lu et al. (2020) and Guo et al. (2021), the adoption intention of farmers’ straw returning technology is characterized from two aspects: adoption intention and promotion intention. The measurement items are based on Likert’s 5-point scale, and the answer options for all questions are “very low,” “lower,” “average,” “higher,” and “very high,” which are assigned “1–5,” respectively. Referring to the research of Cao et al. (2020) and Srisopaporn et al. (2015), the adoption behavior of farmers’ straw returning technology is characterized by two aspects: whether it is adopted or not and the intensity of adoption. Whether or not to adopt the binary valuation method, if the farmer adopts the valuation as 1, the farmer fails to adopt the valuation as 0, and the adoption intensity refers to the number of years that the farmer continuously adopts the straw returning technology.
2. Behavioral attitude, subjective norm, perceived behavioral control, and government support. The measurement items of the four latent variables are all based on Likert’s 5-scale, and the answer options of all questions are “completely disagree,” “disagree,” “basically agree,” “comparatively agree,” and “completely agree,” which are assigned values of 1–5, respectively. Among them, the measurement items of behavioral attitude mainly refer to the research of Li et al. (2020), Bayard and Jolly (2007), and Luzar and Diagne (1999); the measurement items of subjective norm mainly refer to the research of Ajzen (1985) and Beedell and Rehman (2000); the measurement items of perceived behavioral control mainly refer to the research of Ajzen (1985) and Zhang et al. (2021); and the measurement items of government support mainly refer to the research of Zhang and Bin (2018) and Zhang et al. (2021). The specific definitions of variables and measurement items are shown in Table 2.

**Table 2:** Variable definition and measurement items.

Latent variable	Index	Measurement item	Source
Behavioral response (BR)	Whether to adopt	I adopted the straw returning technology (BR_1_)	Cao et al. (2020), Srisopaporn et al. (2015)
	Adoption intensity	Years of continuous adoption of straw returning technology (BR_2_)	
Behavioral intention (BI)	Adoption intention	The degree of my intention to adopt the straw returning technology (BI_1_)	Lu et al. (2020), Guo et al. (2021)
	Promotion intention	The degree of my intention to recommend the straw returning technology to others (BI_2_)	
Behavioral attitude (BA)	Economic benefits	I think straw returning technology can increase grain output and raise income level (BA_1_)	Li et al. (2020), Bayard and Jolly (2007), Luzar and Diagne (1999)
	Social benefit	I think straw returning technology can conducive to rural development and social progress (BA_2_)	
	Ecological benefits	I think straw returning technology can improve ecological environment and rational utilization of resources (BA_3_)	
Subjective norm (SN)	Mandatory norm	Village cadres strongly advocate the adoption of straw returning technology (SN_1_)	Ajzen (1985), Beedell and Rehman (2000)
	Exemplary norm	The social atmosphere of adopting straw returning technology is better (SN_2_)	
Perceived behavioral control (PBC)	Self efficacy	I can master the relevant knowledge and skills (PBC_1_)	Zhang et al. (2021), Ajzen (1985)
		I can bear the economic cost of straw returning technology (PBC_2_)	
	Perceived difficulty	I think straw returning technology is not difficult (PBC_3_)	
		I think the active adoption of straw returning technology will be successful (PBC_4_)	
Government support (GS)	Policy support	Government has provided policy support for straw returning technology (GS_1_)	Zhang and Bin (2018), Zhang et al. (2021)
		Government has provided share straw returning technology experience (GS_2_)	
	Technical support	Government has provided equipment and technical support (GS_3_)	
		Government has provided relevant consultation or training (GS_4_)	

### Model construction

Since the latent variables set in this paper include multiple observable variables, and some observable variables are difficult to directly observe, the Structural Equation Modeling (SEM) model as a multivariate statistical analysis method has strong adaptability. The advantage of SEM is that it can deal with multiple explanatory variables and explained variables together, and can measure the logical relationship between latent variables and observable variables (Bagheri et al., 2019a). Previous studies have shown that SEM has been widely used in the field of social science, and it is reliable and effective in explaining and predicting farmers’ technology adoption behavior (Lou et al., 2021; Wang et al., 2022). As long as SEM can continuously meet the requirements of scholars, it will continue to flourish (Hershberger, 2003). Therefore, we use SEM to study. Its equation expression is as follows:


(1) η=βη+Γξ+ζ

(2) $$Y=\Lambda_{y}\eta +\varepsilon$$

(3) $$X=\Lambda_{x}\xi +\delta$$

Equation (1) is the structural equation, η is the endogenous latent variable; β is the coefficient of the endogenous latent variable η ; ξ is the exogenous latent variable; Γ is the coefficient of the exogenous latent variable ξ ; andζ represents the residual. Equations (2) and (3) are both measurement equations, Y and X are the observed variable vectors of the endogenous latent variable η and the exogenous latent variable ξ , respectively; $and\;\Lambda_{y}$ and $\Lambda_{x}$ represent the difference between Y on η and X on ξ , respectively. Correlation coefficient matrixes; ε and δ, both represent measurement errors.

## Data analysis and empirical results

### Normality, reliability, and validity test

1. Normality test. Maximum likelihood method is a common parameter estimation method of SEM, which requires that the data must obey multivariate normal distribution. Therefore, the skewness and kurtosis of each measurement item were analyzed by SPSS 20.0 first. The results showed that the absolute value of skewness coefficient of measurement items was between 0. 446 and 1.438, all of which were less than 3, and the absolute value of kurtosis coefficient was between 1.145 and 3.162, all of which were less than 10 (Kline, 1998). Therefore, the data of this study passed the normal distribution test.
2. Reliability test. SPSS 20.0 was used to test the overall reliability of the questionnaire and the reliability of the latent variables. The results showed that the Cronbach’s α of the overall index of the questionnaire was 0.804, the Cronbach’s α of the latent variables was 0.614–0.743, and the combined reliability was 0.721–0.864, both of which were greater than 0.6 (Hair et al., 2020), which indicated that the internal consistency of the latent variables was good.
3. Validity test. The validity test includes two aspects: convergent validity and discriminant validity. KMO and Bartlett’s spherical test were used to analyze the convergent validity. The results showed that the calculated KMO value of the overall index of the questionnaire was 0.828, which was greater than the benchmark value of 0.7 (Zhang et al., 2020), and the Bartlett’s spherical test value was equal to 4510.372, which was significant under the condition of 408 degrees of freedom. The calculated KMO values of all latent variables were greater than the benchmark value of 0.5, and the Bartlett’s spherical test values of all latent variables had reached a significant level, which indicated that the data had good convergent validity. The average extracted variance value AVE and the combined reliability value CR are also used to describe the convergent validity. It is generally considered that the AVE of each factor is greater than 0.5 and the CR is greater than 0.7 (Fornell and Larcker, 1981), indicating that it has good convergent validity. As shown in Table 3, the test results all met the research needs.

**Table 3:** Reliability and convergent validity test.

Latent variable	Cronbach’s α coefficient	CR	AVE	KMO measure	Chi-square test	Significant level
BA	0.658	0.864	0.816	0.657	304.361	0.00
SN	0.623	0.826	0.793	0.757	747.702	0.00
PBC	0.770	0.856	0.778	0.784	1007.635	0.00
GS	0.636	0.788	0.736	0.633	110.028	0.00
BI	0.743	0.721	0.717	0.594	398.773	0.00
BR	0.614	0.793	0.792	0.617	135.447	0.00

BA, Behavioral attitude; SN, Subjective norms; PBC, Perceived behavioral control; GS, Government support; BR, Behavioral response; BI, Behavioral intention.

Heterotrait-Monotrait Ratio (HTMT) was used to analyze the discriminant validity. Usually, the value of HTMT less than 0.85 is considered to have discriminant validity between the two variables (Henseler et al., 2015). All HTMT values (Table 4) were within the standard range, which indicated that the data had good discriminant validity.

**Table 4:** Discriminant validity test.

	BA	SN	PBC	GS	BI	BR
BA						
SN	0.493					
PBC	0.601	0.598				
GS	0.590	0.524	0.757			
BI	0.509	0.563	0.486	0.478		
BR	0.498	0.498	0.503	0.472	0.487	

BA, Behavioral attitude; SN, Subjective norms; PBC, Perceived behavioral control; GS, Government support; BR, Behavioral response; BI, Behavioral intention.

### Model fitness test

The purpose of the overall fitness test of the model is to verify whether the relationship hypothesis among the potential variables is reasonable or not, and whether the measure items of the potential variables can fully represent the comprehensive reliability of the potential variables and the research scale. In this paper, the structural equation model was fitted with Amos 24.0. According to the fitting theory of evaluation model, absolute fitting index (X^2^/DF, GFI, AGFI, RMSEA, and SRMR), relative fitting index (NFI, CFI, TLI, and IFI), and reduced fitting index (PNFI, PCFI, and PGFI) were selected to analyze the fitting effect of evaluation model. The model has good fitness when the following conditions are met: X^2^/DF between 1.0 and 3.0, RMSEA and SRMR should be less than 0.08, NFI, CFI, TLI, and IFI should be greater than 0.90, and PNFI, PCFI, and PGFI should be greater than 0.50 (Schreiber et al., 2006; Bagheri et al., 2019b). The results showed (Table 5) that X^2^/DF = 1.563, RMSEA = 0.034, SRMR = 0.021, NFI = 0.927, CFI = 0.959, TLI = 0.946, IFI = 0.961, PNFI = 0.725, PCFI = 0.760, and PGFI = 0.701. They met the criteria, which indicated that the model had good fitness.

**Table 5:** Fitting results of model fitness.

Fitting index	Evaluation index	Reference value	Modified model fitting value	Test result
Absolute fitting index	X^2^/DF	1.0–3.0	1.563	Ideal
	GFI	>0.90	0.976	Ideal
	AGFI	>0.90	0.972	Ideal
	RMSEA	<0.08	0.034	Ideal
	SRMR	<0.08	0.021	Ideal
Relative fitting index	NFI	>0.90	0.927	Ideal
	CFI	>0.90	0.959	Ideal
	TLI	>0.90	0.946	Ideal
	IFI	>0.90	0.961	Ideal
Reduced fitting index	PNFI	>0.50	0.725	Ideal
	PCFI	>0.50	0.760	Ideal
	PGFI	>0.50	0.701	Ideal

### Structural equation model estimation result

**Figure 4:**
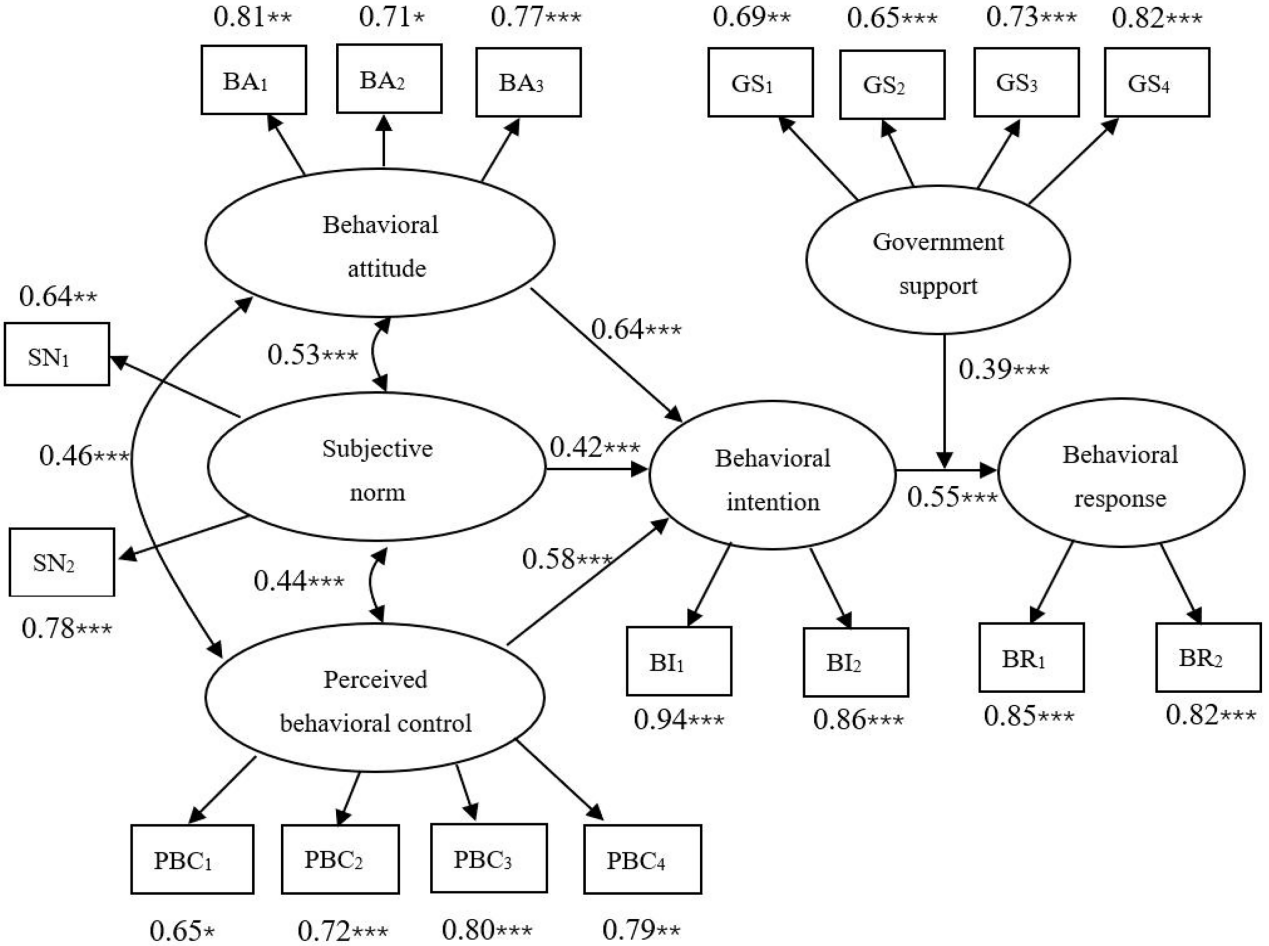
Structural equation model and standardized path coefficient diagram. *p < 0.10, **p < 0.05, ***p < 0.01. BA, Behavioral attitude; SN, Subjective norms; PBC, Perceived behavioral control; GS, Government support; BR, Behavioral response; BI, Behavioral intention.

The model calculation results show (Figure 4) that the behavior logic of farmers’ straw returning technology follows the improved TPB, and the six hypotheses H1–H6 are confirmed.

1. Behavioral attitude. The behavioral attitude of farmers has a direct driving effect on the adoption intention of straw returning technology, and its path coefficient is 0.64. Among the three cognitive factors of farmers’ straw returning technology adoption intention, the path coefficient is the largest, indicating that behavioral attitude is the main cognitive factor driving farmers’ adoption intention of straw returning technology. Among the three observation variables of behavioral attitude, the path coefficients of economic benefit, social benefit, and ecological benefit were 0.81, 0.71, and 0.77, respectively, indicating that positive cognition of straw returning technology benefit can improve farmers’ adoption intention. Compared with social and ecological benefits, farmers pay more attention to economic benefits, which is in line with economic laws, that is, as a rational economic person, the adoption of straw returning technology is a decision made by farmers based on the consideration of profit maximization.
2. Subjective norm. Farmers’ subjective norm has a direct driving effect on the adoption intention of straw returning technology, and its path coefficient is 0.42. Among the three cognitive factors of farmers’ straw returning technology adoption intention, the path coefficient is the smallest, indicating that subjective norm is the effective cognitive factor driving farmers’ adoption intention of straw returning technology. Among the two observed variables of subjective norm, the path coefficients of mandatory norm and exemplary norm are 0.64 and 0.78, respectively, which indicates that farmers will be influenced and pressured by village cadres, relatives, and friends. In contrast, the influence from relatives and friends is greater. In rural China, family is the basic unit of production, and the contact between relatives and friends is generally greater than that with village cadres. Farmers are very concerned about their views on their production behavior, so the adoption behavior of straw returning technology by relatives and friends is more likely to stimulate farmers’ herd mentality and improve their implementation intention.
3. Perceived behavioral control. Farmers’ perceived behavioral control has a direct driving effect on the adoption intention of straw returning technology, and its path coefficient is 0.58. Among the three cognitive factors of farmers’ straw returning technology adoption intention, the path coefficient is larger, indicating that perceived behavioral control is the important cognitive factor driving farmers’ adoption intention of straw returning technology. Among the four observed variables of perceived behavioral control, the path coefficients (0.80, 0.79) of the two observed variables (PBC_3_, PBC_4_) representing perceived difficulty were greater than those (0.65, 0.72) of the two observed variables (PBC_1_, PBC_2_) representing self-efficacy, indicating that perceived difficulty plays a greater role than self-efficacy. Although straw returning technology has been promoted for many years in the surveyed areas, it has not been widely adopted by farmers. Many farmers report that due to natural conditions, technical defects, and other practical problems, straw returning is time-consuming and laborious, and it is difficult to achieve the desired effect, thus reducing the enthusiasm for adoption.
4. Behavioral intention. On the one hand, the behavioral intention of farmers has a direct driving effect on the behavioral response, and its path coefficient is 0.55, indicating that the stronger the behavioral intention is, the more likely the farmers are to adopt the straw returning technology; on the other hand, the three latent variables of subjective cognition are all through the effect of behavioral intention on farmers’ adoption behavior of straw returning technology, it shows that behavioral intention plays a mediating role between subjective cognition and behavioral response.
5. The relationship between behavioral attitude, subjective norm, and perceived behavioral control. There are interaction effects among the three latent variables. The influence coefficient of behavioral attitude and subjective norm path is 0.53, indicating that the clearer the village cadre’s proposition, the more obvious the demonstration of relatives and neighbors, the stronger the call and driving force, and the more positive the behavioral attitude of farmers. Farmers’ positive behavioral attitude will encourage village cadres to actively promote straw returning technology, and at the same time promote sharing and demonstration among relatives and neighbors, thus enhancing subjective norms. The influence coefficient of behavioral attitude and perceptual behavior control path is 0.46, indicating that the stronger the perceptual behavioral control of farmers over straw returning technology. The more positive the behavioral attitude of farmers, the stronger their of perception behavioral control of straw returning technology. The influence coefficient of subjective norm and perceptual behavior control path is 0.44, indicating that the stronger the farmers feel the village cadre’s proposition and the demonstration of relatives and neighbors, the stronger their perceptual behavior control ability; the stronger perceptual behavior control ability of farmers, will also promote village cadres strengthen guidance and actively share with relatives and neighbors, thereby enhancing subjective norms.
6. Government support. Government support has a moderating role in the path from farmers’ behavioral intention to behavioral response, and its influence coefficient is 0.39, indicating that the greater the government support, the more likely the farmers’ behavioral intention will be transformed into actual behavior. Among the four observation variables of government support, the path coefficients (0.73, 0.82) of the two observation variables (GS_3_, GS_4_) representing technical support are larger than the path coefficients (0.69, 0.65) of the two observation variables (GS_1_, GS_2_) representing policy support, indicating that the role of technical support is greater than that of policy support. Therefore, the effective implementation of straw returning technology needs to be equipped with professional and technical personnel to provide consulting and guidance services in each link, answer questions and doubts for farmers in time, enhance farmers’ confidence in technology mastery, and promote their intention to turn into action.

### Multi-group model test

In view of the heterogeneity within the farmer group, this paper uses a multi-group SEM model from the perspective of concurrent occupation to further test the differences in the driving effect of different types of farmers’ subjective cognition on the adoption behavior of straw returning technology. Referring to relevant research (Liu et al., 2021), it is defined that the proportion of farmers’ non-agricultural income in total household income is less than 10% as pure agricultural type, 10–50% as concurrent occupation type I, 50–90% as concurrent occupation type II, and more than 90% as non-agricultural type.

The model test results show that the maximum value of RMESA is 0.070; the minimum values of GFI, NFI, and CFI are 0.932, 0.908, and 0,944, respectively, and the *p* value of the chi-square statistic does not reach a significant level, indicating that the multi-group analysis model has a good fit with the sample data. It can be seen from Table 6 that there are similarities between the analysis results of multi-group samples and full samples: There is an interaction effect among behavioral attitude, subjective norm, and perceived behavioral control, and the three latent variables have a significant driving effect on behavioral intention; behavioral intention plays a mediating role between subjective cognition and behavioral response; government support plays a moderating role in the path from farmers’ behavior intention to behavior response, that is, hypothesis H1–H6 has been verified again in different types of farmers. But there are also some differences, mainly in: (1) The driving intensity of the three latent variables on behavioral intention is significantly different among different types of farmers. The driving path coefficients of behavioral attitude to the behavioral intention of pure agriculture type, concurrent occupation type I, concurrent occupation type II, and non-agricultural type are 0.46, 0.42, 0.33, and 0.31, respectively; the drive path coefficients of subjective norm are 0.34, 0.29, 0.21, and 0.19, respectively; and the drive path coefficients of perceptual behavioral control are 0.32, 0.29, 0.28, and 0.22, respectively. This indicates that the driving effect of subjective cognition on farmers’ intention to adopt straw returning technology weakens with the increase of the degree of concurrent occupation. The possible reason is that farmers with a lower degree of concurrent occupation are more dependent on land. The survey found that the pure agricultural type and concurrent occupation type I are more fully aware of the positive benefits of straw returning, have less difficulty in perceiving behavioral ability, and have stronger behavioral intention. However, farmers with a high degree of concurrent occupation mainly derive their income from non-agricultural activities, pay less attention to the productivity and sustainable utilization of cultivated land, and their behavioral intention is not high because of the awareness of the lost work caused by adopting straw returning technology. (2) Government support can prompt the four types of farmers to transform their behavioral intention into actual behavior, but the intensity of its influence gradually weakens with the increase of farmers’ concurrent occupation. On the one hand, government support can effectively reduce the risk and transaction cost of farmers adopting straw returning, and provide farmers with technical and management guarantees. On the other hand, the adoption of individual behavior by farmers to return straw has risen to collective action to achieve the improvement of collective welfare, which requires the incentive and regulation of government systems and policies. Therefore, government support has a positive intervention effect on farmers’ behavioral responses, but this intervention effect will gradually weaken with the reduction of farmers’ holdings of agricultural production means and the weakening of their links with rural social networks.

**Table 6:** Estimation results of multi-group model test.

Path	Pure agricultural type		Concurrent occupation type I		Concurrent occupation type II		Non-agricultural type	
	Estimate	S.E.	Estimate	S.E.	Estimate	S.E.	Estimate	S.E.
BI ← BA	0.46***	3.32	0.42***	3.67	0.33**	5.22	0.31**	4.18
BI ← SN	0.34**	3.62	0.29**	3.07	0.21*	4.25	0.19*	1.86
BI ← PBC	0.32**	2.66	0.29***	5.29	0.28**	3.74	0.22**	4.31
BR ← BI	0.43***	4.45	0.37***	3.52	0.30**	2.87	0.27*	1.64
BA ↔ SN	0.13**	1.79	0.24*	3.21	0.35***	4.93	0.37***	4.76
SN ↔ PBC	0.08*	2.86	0.16*	4.03	0.24**	5.19	0.26***	4.64
BA ↔ PBC	0.11**	2.77	0.20**	3.83	0.21**	5.84	0.26***	5.19
GS ← BI	0.43**	2.45	0.36**	3.15	0.24**	3.22	0.19**	2.74
BR ← GS	0.44***	3.02	0.38**	2.99	0.25**	3.61	0.21*	3.26

BA, Behavioral attitude; SN, Subjective norms; PBC, Perceived behavioral control; GS, Government support; BR, Behavioral response; BI, Behavioral intention.

* *p* < 0.10;

** *p* < 0.05;

*** *p* < 0.01.

## Conclusion and suggestions

### Conclusion

Guided by the improved TPB, this paper uses the multi-group SEM model to analyze the driving mechanism of subjective cognition on the adoption behavior of farmers’ straw returning technology, and draws the following main conclusions:

Farmers’ adoption behavior of straw returning technology follows the driving path of “cognition → intention → behavior,” and three latent variables, which represent subjective cognition, such as behavioral attitude, subjective norm, and perceived behavioral control, have significant driving effects on farmers’ adoption intention of straw returning technology, and behavioral intention plays a mediating role in the process of subjective cognition driving farmers’ adoption behavior of straw returning technology. The driving effect of the three latent variables of subjective cognition on the farmers’ straw returning technology adoption intention is behavioral attitude, perceived behavioral control, and subjective norm in descending order, and there is an interaction effect among the three. Economic benefits play the largest role in behavioral attitudes, followed by ecological benefits and social benefits; the role of exemplary norm in subjective norm is greater than that of mandatory norm; the role of perceived difficulty in perceived behavior control is greater than self-efficacy. Government support plays a moderating role from farmers’ behavioral intention to behavioral response, and the role of technical support is greater than that of policy support. The subjective cognition of different types of farmers has a significant driving effect on the adoption of straw returning technology, but the driving strength weakens with the increase of the degree of farmers’ concurrent occupation.

### Suggestions

Based on the study, this paper puts forward the following suggestions:

1. Make full use of television broadcasts, online media, publicity manuals, and other forms to increase the publicity and interpretation of relevant policies, improve farmers’ awareness of economic benefits, and enhance farmers’ confidence in the technical and economic prospects of straw returning.
2. Accelerate the construction of typical demonstration models, give full play to the demonstration and driving role of relatives and friends, and enable farmers to “learn by doing,” and create a strong and positive social atmosphere.
3. Increase the training, guidance, and service of straw returning technology; improve the scope and standards of subsidies; ease farmers’ cognition of restrictive conditions and enhance farmers’ behavioral ability; and make them truly feel the economic benefits of straw returning technology.
4. The promotion of straw returning technology needs “classified and precise policy.” For farmers with a low degree of concurrent occupation, they should strengthen their awareness of environmental responsibility and give further preferential policy support; for farmers with a high degree of concurrent occupation, the circulation and trusteeship of cultivated land can be encouraged to promote the effective utilization of straw.

### Limitations and future research

This study also has certain limitations: Firstly, the study randomly selected four provinces in China. The scale of the study object is relatively narrow, the research results may not be directly extended to other parts of China, and the study area needs to be expanded in the future. Secondly, the study focuses on the analysis of the influence of subjective cognition on farmers’ straw returning behavior, but does not cover all the factors that affect farmers’ straw returning behavior, the factors need to be further supplemented in the future.

## Data availability statement

The original contributions presented in the study are included in the article/Supplementary material, further inquiries can be directed to the corresponding author.

## Author contributions

ZR: writing—original draft. KZ: reviewing and editing. All authors contributed to the article and approved the submitted version.

## Conflict of interest

The authors declare that the research was conducted in the absence of any commercial or financial relationships that could be construed as a potential conflict of interest.

## Publisher’s note

All claims expressed in this article are solely those of the authors and do not necessarily represent those of their affiliated organizations, or those of the publisher, the editors and the reviewers. Any product that may be evaluated in this article, or claim that may be made by its manufacturer, is not guaranteed or endorsed by the publisher.
