# Supplementary material for: Driving mechanism of subjective cognition on farmers’ adoption behavior of straw returning technology: Evidence from rice and wheat producing provinces in China
Source: Front Psychol. 2022 Aug 2;13:922889. doi: 10.3389/fpsyg.2022.922889 (PMC9379131; doi:10.3389/fpsyg.2022.922889)
Supplement: Supplementary file 1 [file Data_Sheet_1.docx]

**Appendix 1. Questionnaire**

**Survey on the adoption behavior of farmers' straw returning technology**

City County Town Village Date

**A. Farmers and their families situation in 2021**

A1. Gender ( )

1= Female; 2= Male

A2. Age

A3. Education level ( )

1= primary school; 2= junior high school; 3=high school; 4= junior college; 5= University and above

A4. Planting years

A5. Health condition ( )

1=very poor; 2=poor; 3=fair; 4=good; 5=very good

A6. Planting area (Mu)

A7. Number of household labor

A8. Annual income/ million RMB

**B. Farmers' adoption and cognition of straw returning technology in 2021**

**B1. The degree of my intention to adopt the straw returning technology ( )**

1= very low; 2= lower; 3= average; 4= higher; 5= very high

**B2. The degree of my intention to recommend the straw returning technology to others ( )**

1= very low; 2= lower; 3= average; 4= higher; 5= very high

**B3. I adopted the straw returning technology ( )**

1= Yes; 2= No

**B4. Years of continuous adoption of straw returning technology**

**B5. I think straw returning technology can increase grain output and raise income level ( )**

1= completely disagree; 2= disagree; 3= basically agree; 4= comparatively agree; 5= completely agree

**B6. I think straw returning technology can conducive to rural development and social progress ( )**

1= completely disagree; 2= disagree; 3= basically agree; 4= comparatively agree; 5= completely agree

**B7. I think straw returning technology can improve ecological environment and rational utilization of resources ( )**

1= completely disagree; 2= disagree; 3= basically agree; 4= comparatively agree; 5= completely agree

**B8. Village cadres strongly advocate the adoption of straw returning technology ( )**

1= completely disagree; 2= disagree; 3= basically agree; 4= comparatively agree; 5= completely agree

**B9. The social atmosphere of adopting straw returning technology is better ( )**

1= completely disagree; 2= disagree; 3= basically agree; 4= comparatively agree; 5= completely agree

**B10. I can master the relevant knowledge and skills ( )**

1= completely disagree; 2= disagree; 3= basically agree; 4= comparatively agree; 5= completely agree

**B11. I can bear the economic cost of straw returning technology ( )**

1= completely disagree; 2= disagree; 3= basically agree; 4= comparatively agree; 5= completely agree

**B12. I think straw returning technology is not difficult ( )**

1= completely disagree; 2= disagree; 3= basically agree; 4= comparatively agree; 5= completely agree

**B13. I think the active adoption of straw returning technology will be successful ( )**

1= completely disagree; 2= disagree; 3= basically agree; 4= comparatively agree; 5= completely agree

**B14. Government has provided policy support for straw returning technology ( )**

1= completely disagree; 2= disagree; 3= basically agree; 4= comparatively agree; 5= completely agree

**B15. Government has provided share straw returning technology experience ( )**

1= completely disagree; 2= disagree; 3= basically agree; 4= comparatively agree; 5= completely agree

**B16. Government has provided equipment and technical support ( )**

1= completely disagree; 2= disagree; 3= basically agree; 4= comparatively agree; 5= completely agree

**B17. Government has provided relevant consultation or training ( )**

1= completely disagree; 2= disagree; 3= basically agree; 4= comparatively agree; 5= completely agree
